# Inverse Regulation of EGFR/HER1 and HER2-4 in Normal and Malignant Human Breast Tissue

**DOI:** 10.1371/journal.pone.0074618

**Published:** 2013-08-22

**Authors:** Marianne Hauglid Flågeng, Stian Knappskog, Ben P. Haynes, Per Eystein Lønning, Gunnar Mellgren

**Affiliations:** 1 Department of Clinical Science, University of Bergen, Bergen, Norway; 2 Hormone Laboratory, Haukeland University Hospital, Bergen, Norway; 3 Department of Oncology, Haukeland University Hospital, Bergen, Norway; 4 Academic Biochemistry, Royal Marsden Hospital, London, United Kingdom; University of South Alabama, United States of America

## Abstract

Cross-talk between the estrogen and the EGFR/HER signalling pathways has been suggested as a potential cause of resistance to endocrine therapy in breast cancer. Here, we determined HER1-4 receptor and neuregulin-1 (NRG1) ligand mRNA expression levels in breast cancers and corresponding normal breast tissue from patients previously characterized for plasma and tissue estrogen levels. In tumours from postmenopausal women harbouring normal *HER2* gene copy numbers, we found *HER2* and *HER4*, but *HER3* levels in particular, to be elevated (2.48, 1.30 and 22.27 –fold respectively; *P*<0.01 for each) compared to normal tissue. Interestingly, *HER3* as well as *HER4* were higher among ER+ as compared to ER- tumours (*P*=0.004 and *P*=0.024, respectively). *HER2* and *HER3* expression levels correlated positively with ER mRNA (*ESR1*) expression levels (r=0.525, *P*=0.044; r=0.707, *P*=0.003, respectively). In contrast, *EGFR/HER1* was downregulated in tumour compared to normal tissue (0.13-fold, *P*<0.001). In addition, *EGFR/HER1* correlated negatively to intra-tumour (r=-0.633, *P*=0.001) as well as normal tissue (r=-0.556, *P*=0.006) and plasma estradiol levels (r=-0.625, *P*=0.002), suggesting an inverse regulation between estradiol and *EGFR/HER1* levels. In ER+ tumours from postmenopausal women, *NRG1* levels correlated positively with *EGFR/HER1* (r=0.606, *P*=0.002) and negatively to *ESR1* (r=-0.769, *P*=0.003) and E2 levels (r=-0.542, *P*=0.020). Our results indicate influence of estradiol on the expression of multiple components of the HER system in tumours not amplified for HER2, adding further support to the hypothesis that cross-talk between these systems may be of importance to breast cancer growth *in vivo*.

## Introduction

Breast cancer is the most frequent cancer among women world-wide. Estradiol (E2) stimulation through the estrogen receptor (ER) and constitutional HER2 proto-oncogene hyperactivity represent two pivotal pathways regulating breast cancer growth. Breast cancers expressing the ER in general belongs to either the luminal A or B sub-class, each characterized by a distinct gene expression profile [1]. Moreover, between 16 and 20% of all breast cancers are amplified for the HER2 proto-oncogene; these tumours in general belong to the so-called “HER2” class, again characterized by a distinct gene expression profile [1]. The importance of ER and HER2 activation to breast cancer growth is underlined by improved outcome for patients with advanced ER+ and *HER2* amplified early breast cancers treated with an aromatase inhibitor and anti-HER2 therapy in concert [2,3].

Similar to what has been recorded for chemotherapy, *de novo* and acquired drug resistance become the main obstacles to cure by anti-hormonal as well as anti-HER2 therapies. Cross-talk between the HER2- and ER-downstream gene activation pathways has been suggested as a cause of endocrine resistance [4]. Notably, two studies [2,3] revealed improved time to progression for ER+ HER2 amplified metastatic breast cancers having either trastuzumab or lapatinib added to treatment with an aromatase inhibitor. However the benefit of HER2 targeting treatment in *HER2* non-amplified breast cancer is not well known. *In vitro* models of acquired endocrine resistance show modest upregulation of HER2 [5,6] and, interestingly, lapatinib has been shown to restore endocrine sensitivity in these cell lines [5]. Moreover, previous data from our group indicate that HER2 may be upregulated during estrogen deprivation in breast tumours [7]. Further, experimental evidence has implicated EGFR/HER1 as well as HER3 and HER4 to endocrine resistance [8,9]. In general, ER and HER2 positivity are known to be inversely correlated [10–12]. However, while most ER+ tumours are non-amplified for *HER2*, nearly 50% of all *HER2* amplified tumours express ER at moderate to low concentrations [13,14], and recently, HER2 and ER have been shown to be positively correlated in *HER2* non-amplified tumours [15]. Taken together, these findings indicate a potential role for HER2 as well as other components of the HER-receptor family in *HER2* non-amplified breast cancer and endocrine resistance [16].

In this study, we aimed to further explore potential associations between estrogen levels and expression levels of the HER-family members in *HER2* non-amplified breast cancer and in normal breast tissue. To do so, we analysed *HER-1–4* and ligand *NRG1* mRNA expression pattern in breast cancer and normal breast tissue and correlated findings to ER mRNA expression (*ESR1*) and plasma, normal tissue and breast cancer tissue estrogen levels previously determined [17,18]. Our findings add novel information, and provides a better understanding of the potential interactions in-between members of the HER system and their regulation by estradiol.

## Materials and Methods

### Ethics Statements

The study was presented and exempted from review by the Regional Committee for Medical and Health Research Ethics (REK) at the time of collection. All patients provided written informed consent, and the study was performed in accordance to Norwegian law and regulations. After the samples had been collected, each patient was allocated a trial number, demographic data collected, and the database anonymised.

### Study population and sample collection

The breast cancer patients included in this study (n = 42) have been described previously [18]. In short, pre- and postmenopausal women with ER+ or ER- breast cancer, selected for mastectomy at the Department of Surgery, Haukeland University Hospital, Bergen, Norway were included. Patients that had taken any hormone replacement therapy within the 4 weeks pre-surgical period were excluded. Tissues obtained from mastectomy specimens, both normal and tumour tissue, were removed and immediately snap-frozen in liquid nitrogen in the operating theatre, before storage in liquid nitrogen until use. Normal tissue was isolated from the breast quadrant farthest from the tumour-containing quadrant in the breast. Blood samples for plasma measurements were obtained at the day of surgery after fasting overnight, and stored at -20^°^C until use. Normal tissue was available from all but one patient and tumour tissues were available from all but two other patients, leaving 39 patients for statistical comparisons between tumour and normal tissue.

### Multiplex Ligation-dependent Probe Amplification (MPLA)

Gene-amplifications of *EGFR/HER1* and *HER2* were analysed by MLPA using the SALSA MLPA Breast tumour kit (P078-B1; MRC-Holland, Amsterdam, The Netherlands) according to the manufacturer’s instructions. In the patient samples, the peak areas of all MLPA products resulting from *EGFR/HER1* and *HER2* specific probes were first normalized by the average of peak areas resulting from control probes specific for locations outside of chromosomes 7/17. A ratio was then calculated where this normalized value was divided by the corresponding value from a sample consisting of pooled DNA from 10 healthy individuals. A sample was scored as having a reduced copy number at a specific location if this ratio was below 0.75, and as having an increased copy number if the ratio was above 1.25.

### Real time PCR quantification

Total RNA was extracted from ~25 mg tissue using Trizol (Invitrogen, Carlsbad, CA) according to the manufacturer’s recommendations. The RNA was re-suspended in PCR-grade water and concentrations were estimated by optical density (OD) measurement using the Nanodrop (Saveen Werner, Copenhagen, Denmark). For each sample, 1 μg total RNA was reversely transcribed by the 1^st^ Strand cDNA Synthesis Kit (Roche, Basel, Switzerland) using random primers. The cDNA was diluted 1/10 in PCR-grade water and stored at -20^°^C until use.

Real time PCR analyses were performed in three parallel runs on a Light Cycler 480 (LC480) thermo cycler (Roche, Basel, Switzerland) and a negative control without any cDNA was included in each run. Gene specific primers and probes were designed using the Universal Probe Library (UPL, Roche, Basel, Switzerland), and all analyses were run in duplex with the TATA-box binding protein (TBP) reference analyse kit using the Probe Master kit (Roche, Basel, Switzerland). Assay with primer sequences and UPL probes are given in Table 1. The amplification reaction mixture consisted of 2.5 µL diluted cDNA, 10 µL LC480 Probe Master mix, 0.4 µmol/L of each target primer, 0.2 µmol/L of target UPL probe, 0.2 µmol/L of TBP reference primers and 0.1 µmol/L TBP reference probe in a total volume of 20µL. Termocycling conditions used were pre-incubation at 95 ^°^C for 10 minutes followed by 45 cycles with denaturation at 95 ^°^C for 10 seconds, primer annealing at 60 ^°^C for 30 seconds and DNA sequence extension at 72 ^°^C for 1 second followed by fluorescence measurement. The PCR products were then cooled at 40 ^°^C. Crossing points (Cp) for both target gene and TBP and the efficiency from standard curves from a serially diluted cDNA sample were used to quantify relative expression levels of each target gene separately. The relative mRNA expression levels are presented as the mRNA expression level of target gene divided by the mRNA expression levels of the reference gene TBP in each single sample.

**Table 1:** Primer sequences and UPL probes used for real-time PCR.

**Gene**	**Forward (left) primer**	**Reverse (right) primer**	**UPL probe**†
*EGFR/HER1*	5'-cagccacccatatgtaccatc-3'	5'-aactttgggcgactatctgc-3'	42
*HER2*	5'-ccctgacctgctggaaaag-3'	5'-ggccgacattcagagtcaat-3'	43
*HER3*	5'-acagccccagatctgcac-3'	5'-gttgggcgaatgttctcatc-3'	9
*HER4*	5'-ttccactttaccacaacatgcta-3'	5'-cagaatgaagagcccacca-3'	78
*NRG1*	5'-gatcagcaaattaggaaatgacag-3'	5'-ggcataccagtgatgatctcg-3'	53

† Universal probe library (UPL) probe number (Roche, Basel, Switzerland).

*ESR1* mRNA expression levels in tumours from 28 (9 pre- and 19 postmenopausal) of the 42 patients have been analysed and reported previously [17].

### Measurement of estrogen levels

Estrogen levels measured in plasma and the matched normal and tumour tissue samples from 13 premenopausal and 29 postmenopausal women have been reported previously [18]. In brief, estrogen fractions were measured with highly sensitive RIA methods subsequent to pre-analytical purification through LH20 column (plasma) or HPLC (tissue) chromatography [19,20]. Sensitivity limits for the different analysis were 1.14 pmol/L for E1, 0.67 pmol/L for E2, and 0.55 pmol/L for E1S [20].

### Statistical analysis

The mRNA expression levels are presented as geometric mean with 95% confidence interval (CI) of the mean. Boxplot and stem-and-leaf plot were used to present median mRNA expression levels, quartiles and outliers within each group. Correlation analyses of the expression of *HER*-receptors and *NRG1* in normal and tumour tissue and the levels of E1, E2 and E1S in normal and tumour tissue and plasma were analysed using the Spearman Rank test. Differences in mRNA expression between related tumour and normal-tissue samples were analysed using the non-parametric Wilcoxon test. Differences in mRNA expression levels between ER+ and ER- or HER2 amplified and non-amplified subjects were analysed using non-parametric Mann-Whitney U rank test of independent samples. All *P*-values are two-sided and the threshold *P*-value for statistical significance was 0.05. All analyses were performed using the software SPSS Statistics version 19 (IBM SPSS Statistics).

## Results

### Patient characteristics and tissue specimens

**Figure 1:**
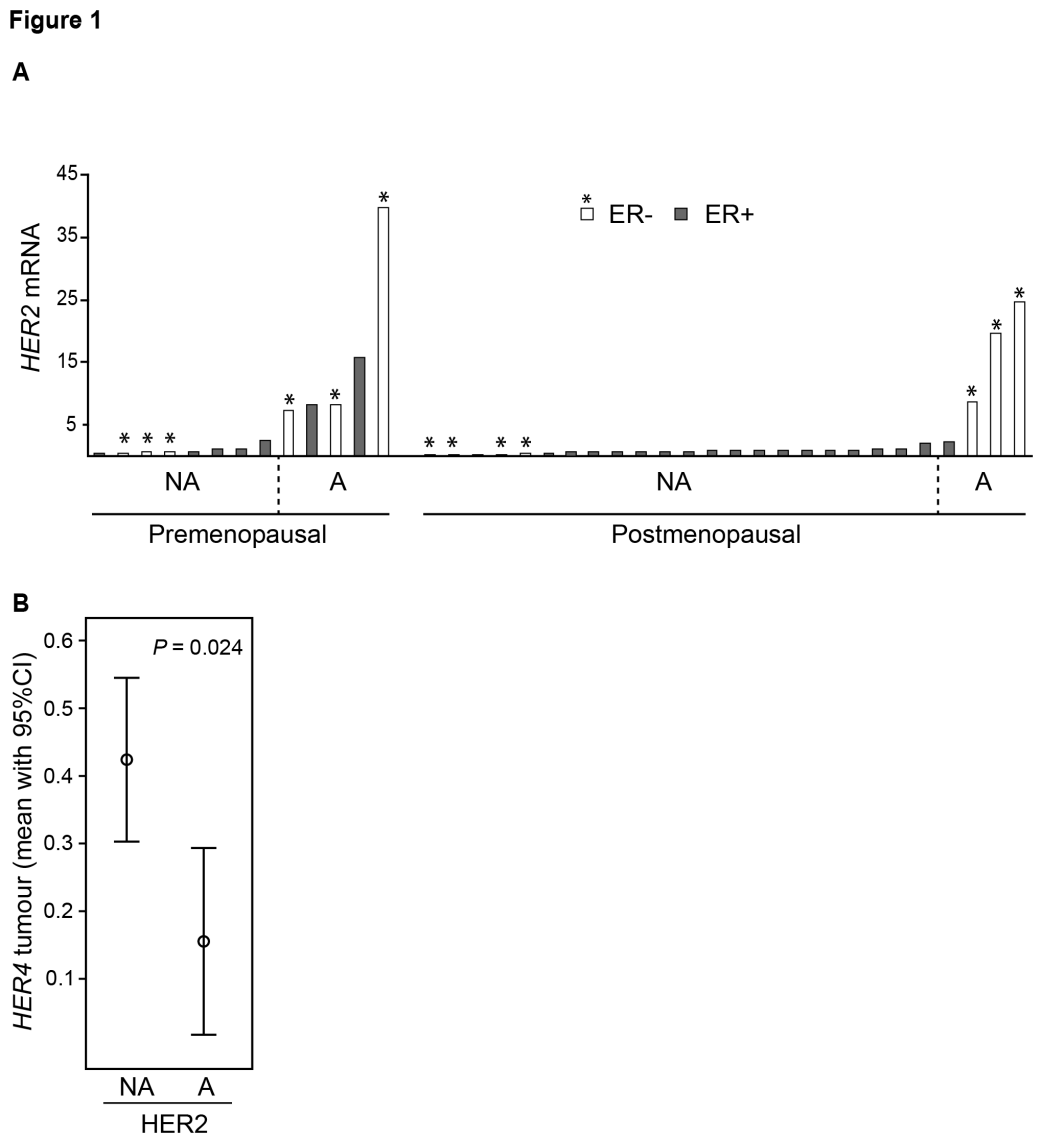
*HER2* tumour levels and classification of *HER2* amplified and non-amplified cancers. A) Relative mRNA expression levels of *HER2* in estrogen receptor positive (ER+; black bars) and ER negative (- ; white bars with asterisk) breast tumours from pre- and postmenopausal women. Tumours identified to be *HER2* non-amplified (NA) and amplified (A) by MPLA are indicated. B) Relative *HER4* levels in *HER2* NA and A tumours. Significant difference between A and NA tumours is presented using the Mann-Whitney U test.

The study population including 42 pre- and postmenopausal breast cancer patients with ER+ and ER- disease (invasive carcinomas) has been described in detail previously (Table 2 [18]). Breast cancer and normal tissue from the same breast were available from 39 of the 42 patients. Nine tumours were amplified (>2 alleles) for the *HER2* gene (range 3-14 alleles) and one tumour was amplified for the *EGFR/HER1* gene. Tumours harbouring an elevated number of HER2 alleles in general presented high mRNA expression levels of *HER2* (Figure 1A). Even though both ER+ (*P*=0.002) and ER- (*P*=0.001) tumours exhibit a significant higher *HER2* mRNA levels in *HER2* amplified compared to non-amplified tumours, the difference in *HER2* mRNA levels between amplified and non-amplified tumours were most evident among ER- tumours (Figure 1A). Interestingly, we also observed a decreased *HER4* mRNA expression in tumours with *HER2* gene amplification compared to non-amplified tumours (*P*=0.024, Figure 1B) suggesting that increased levels of *HER2* may be associated with *HER4* suppression.

**Table 2:** Patient and tumour characteristics.

		**Premenopausal (*n* = 13)**	**Postmenopausal (*n* = 29^a^)**
Median age (range), y		41 (31-49)	61 (44-81)
IHC ER, n	ER+	7	20
	ER-	6	9
IHC PR, n	PR+	7	17
	PR-	6	12
MLPA, *EGFR/HER1*, n	Ampl*	0	1
	Non-ampl	13	25
MLPA, *HER2*, n	Ampl^b^	5	4
	Non-ampl	8	22

Abbreviations: IHC, Immunohistochemistry; ER, estrogen receptor; PR, progesterone receptor

^a^ Normal tissue was not available for MLPA and mRNA analyses from one patient and tumour tissues were not available from two other patients

^b^ More than 2 alleles of either *HER2* or *EGFR* were considered amplified.

### Expression of HER receptors and NRG1 in tumour and normal tissue

Considering *HER2* amplified tumours as a distinct class, we restricted this analysis to tumours harbouring a normal *HER2* copy number. The mRNA expression levels of all analysed genes were found to be log normally distributed except for *EGFR/HER1* in normal tissue and *HER4* in tumour tissue that were found to be normally distributed.

**Figure 2:**
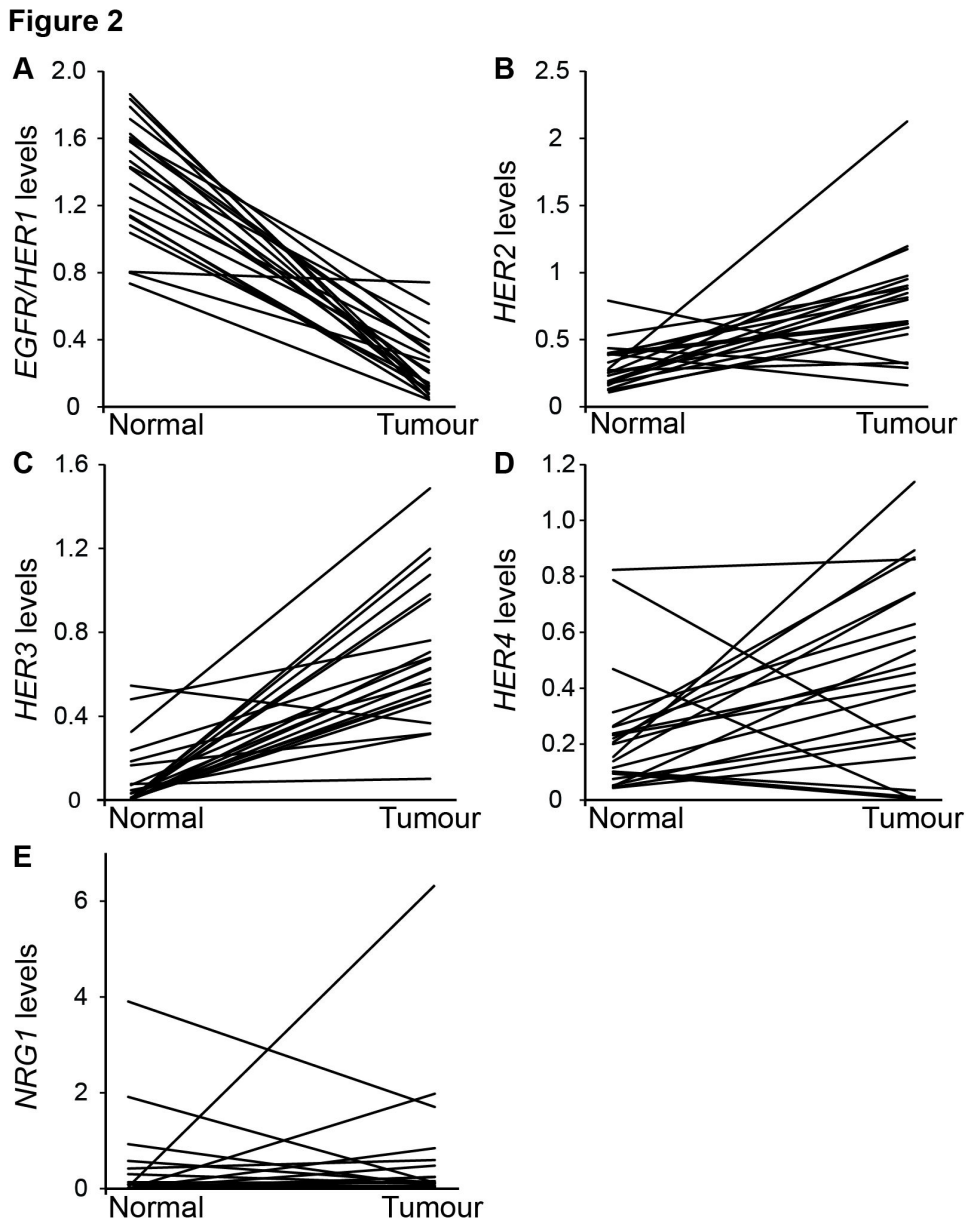
Changes in *HER1-4* and *NRG1* expression levels from normal to tumour breast tissue. Individual mRNA levels of *EGFR/HER1*(A), *HER2* (B), *HER3* (C), *HER4* (D) and *NRG1* (E) from normal and tumour tissue from each postmenopausal patient.

Expression levels of *EGFR/HER1*, *HER2*, *HER3*, *HER4* and *NRG1* in normal and breast cancer tissue are presented in Table 3 and Figure 2. Comparing paired tumour and normal tissue samples, we found a significantly lower level of *EGFR/HER1* in tumour compared to normal tissue both among premenopausal (8 of 8 patients, individual ratio 0.10 (95% CI: 0.047-0.23), *P*=0.012) as well as postmenopausal (22 of 22, individual ratio: 0.13; CI: 0.09-0.20, *P*<0.001) women. In contrast, *HER2* and *HER3* expression levels were higher in tumours compared to normal tissue. Thus, *HER2* was elevated in 7 of 8 premenopausal tumours (individual ratio: 2.64; CI: 1.50-4.63, *P*=0.017) and *HER3* in 8 of 8 individuals (individual ratio: 16.96; CI: 4.02-71.52, *P*=0.012). In postmenopausal tumours, *HER2* was elevated in 19 of 22 (individual ratio: 2.48; CI: 1.70-3.65, *P*<0.001) and *HER3* in 21 out of 22 (individual ratio: 22.27; CI: 9.03-54.92, *P*<0.001). *HER4* expression levels were significantly higher in tumours compared to normal tissue among postmenopausal women only (17 of 22, individual ratio: 1.30 CI: 0.54-3.16, *P*=0.006). No significant difference in *NRG1* levels between normal breast and cancer tissue was recorded.

**Table 3:** *HER1-4* and *NRG1* levels in normal and breast cancer tissue among postmenopausal patients.

**Gene**	**Menopause stage**	**Normal tissue**	**Tumour tissue**	**Fold change (95% CI)**	**Tumour versus normal tissue^a^**	***P* for change^b^**
*EGFR/HER1*	Pre	1.31 (1.07-1.62)^c^	0.14 (0.058-0.32)	0.10 (0.047-0.23)	0↑ 8↓	*P*=0.012
	Post	1.32 (1.17-1.48)	0.18 (0.12-0.26)	0.13 (0.09-0.20)	0↑ 22↓	*P*≤0.001
*HER2*	Pre	0.31 (0.20-0.50)	0.83 (0.52-1.33)	2.64 (1.50-4.63)	7↑ 1↓	*P*=0.017
	Post	0.27 (0.21-0.34)	0.66 (0.52-0.85)	2.48 (1.70-3.65)	19↑ 3↓	*P*≤0.001
*HER3*	Pre	0.062 (0.015-0.26)	1.05 (0.45-2.43)	16.96 (4.02-71.52)	8↑ 0↓	*P*=0.012
	Post	0.027 (0.012-0.061)	0.60 (0.47-0.78)	22.27 (9.03-54.92)	21↑ 1↓	*P*≤0.001
*HER4*	Pre	0.16 (0.10-0.27)	0.25 (0.096-0.67)	1.54 (0.69-3.45)	5↑ 3↓	*P*=0.123
	Post	0.16 (0.11-0.23)	0.23 (0.11-0.50)	1.30 (0.54-3.16)	17↑ 5↓	*P*=0.006
*NRG1*	Pre	0.025 (8.76*10^-4^-0.72)	0.071 (0.031-0.16)	2.86 (0.11-77.45)	4↑ 4↓	*P*=0.263
	Post	0.013 (0.0026-0.062)	0.11 (0.045-0.29)	8.97 (1.90-42.40)	14↑ 8↓	*P*=0.506

^a^ Number of patients with higher (↑) or lower (↓) tumour compared to normal tissue mRNA levels.

^b^ P value from Wilcoxon Signed Ranks test for paired samples.

^c^ mRNA levels presented as geometric mean (95% CI).

*HER3* expression correlated positively to *HER2* (r=0.532, *P*=0.009) as well as *HER4* (r=0.480, *P*=0.020), but negatively to *EGFR/HER1* (r=-0.450, *P*=0.031, Table 4) in tumour tissue from postmenopausal patients. Interestingly we observed a strong positive correlation between intratumour *EGFR/HER1* and *NRG1* levels (r=0.606, *P*=0.002).

**Table 4:** Correlations between HER-receptors and NRG1 in normal and breast tumour tissue among postmenopausal women.

			Normal tissue				Tumour				
			*HER2*	*HER3*	*HER4*	*NRG1*	*EGFR/HER1*	*HER2*	*HER3*	*HER4*	*NRG1*
Normal tissue	*EGFR/HER1*	Total^a^	-0.371	-0.211	-0.197	-0.221	-0.004	0.353	0.307	0.019	0.125
		ER+	-0.183	-0.093	-0.153	-0.154	0.017	0.290	0.222	-0.195	0.082
	*HER2*	Total		0.616**	0.444*	0.681**	0.199	-0.205	-0.398	-0.103	0.106
		ER+		0.527*	0.488*	0.665**	0.084	-0.001	-0.294	0.106	0.162
	*HER3*	Total			0.182	0.817**	0.343	-0.197	-0.269	-0.220	0.091
		ER+			0.212	0.911**	0.232	-0.055	-0.137	0.121	0.189
	*HER4*	Total				0.324	0.135	-0.133	0.193	0.372	0.153
		ER+				0.298	0.096	-0.152	0.216	0.482*	0.127
	*NRG1*	Total					0.318	-0.031	-0.042	-0.038	0.286
		ER+					0.219	0.008	0.004	0.115	0.215
Tumour	*EGFR/HER1*	Total						-0.318	-0.450*	-0.334	0.606**
		ER+						-0.271	-0.321	-0.092	0.744**
	*HER2*	Total							0.532**	0.204	0.076
		ER+							0.564*	0.005	-0.222
	*HER3*	Total								0.480*	-0.144
		ER+								0.183	-0.327
	*HER4*	Total									0.244
		ER+									0.253

^a^ The total group of postmenopausal women and the subgroup of ER+ postmenopausal women.

Spearman rank correlation (two-tailed):

*P≤ 0.05

** *P*≤ 0.01

### Correlations between HER receptors / NRG1 expression levels versus ER-status and plasma and tissue estradiol levels

**Figure 3:**
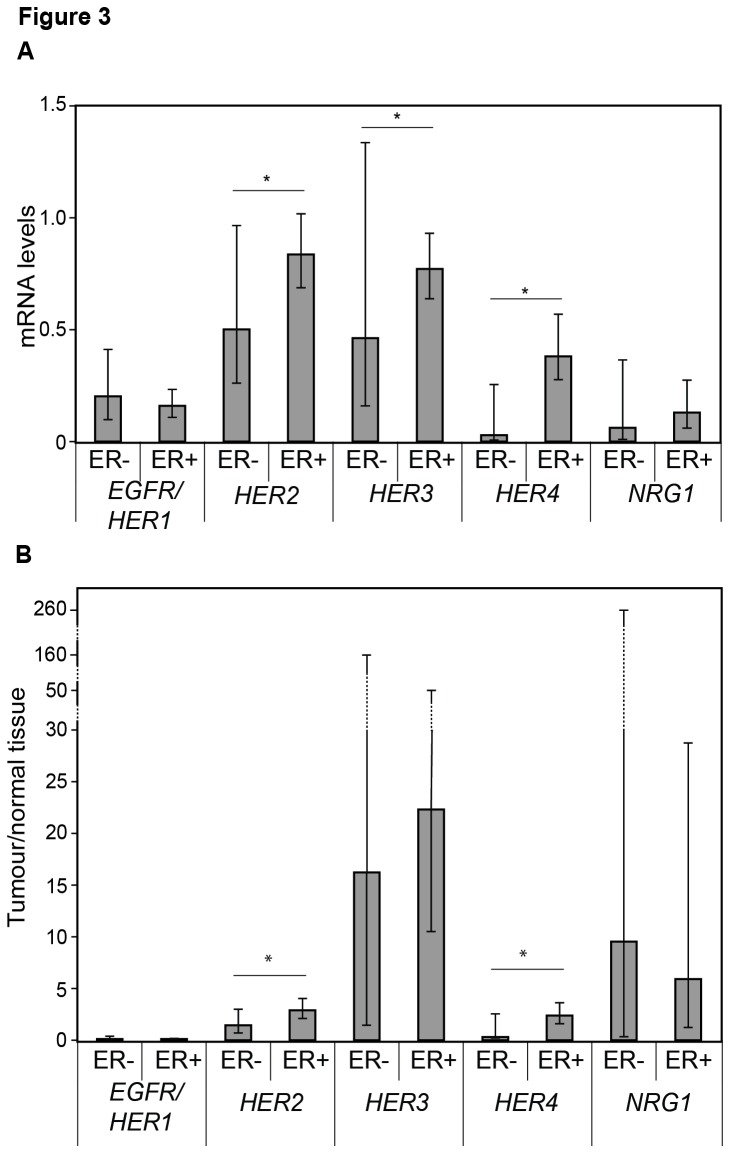
*HER1-4* and *NRG1* levels related to estrogen receptor status. Geometric mean with 95% confidence intervals of the *HER*-receptors and *NRG1* in estrogen receptor positive (ER+) and ER negative (-) tumours (A) and intervals of tumour to normal tissue ratio (B) among all patients with *HER2* non-amplified disease. Significant differences between ER+ and ER- tumours are presented using the Mann-Whitney U test.

Among all *HER2* non-amplified tumours, we found *HER2* (*P*=0.026) in addition to *HER3* (*P*=0.030) and *HER4* (*P*=0.007) to be higher among ER+ as compared to ER- tumours (Figure 3A). Moreover, a higher tumour to normal tissue concentration ratio was observed for *HER2* (*P*=0.042) as well as *HER4* (*P*=0.012) among ER+ tumours as compared to ER- tumours (Figure 3B). In addition, both *HER2* (r=0.547, *P*=0.001, data not shown) and *HER4* (r=0.513, *P*=0.017) correlated positively with *ESR1* expression levels in these *HER2* non-amplified tumours including both pre- and postmenopausal women. *ESR1* mRNA expression levels were obtained from a previous study were high *ESR1* expression levels were associated with ER+ tumours [17].

**Figure 4:**
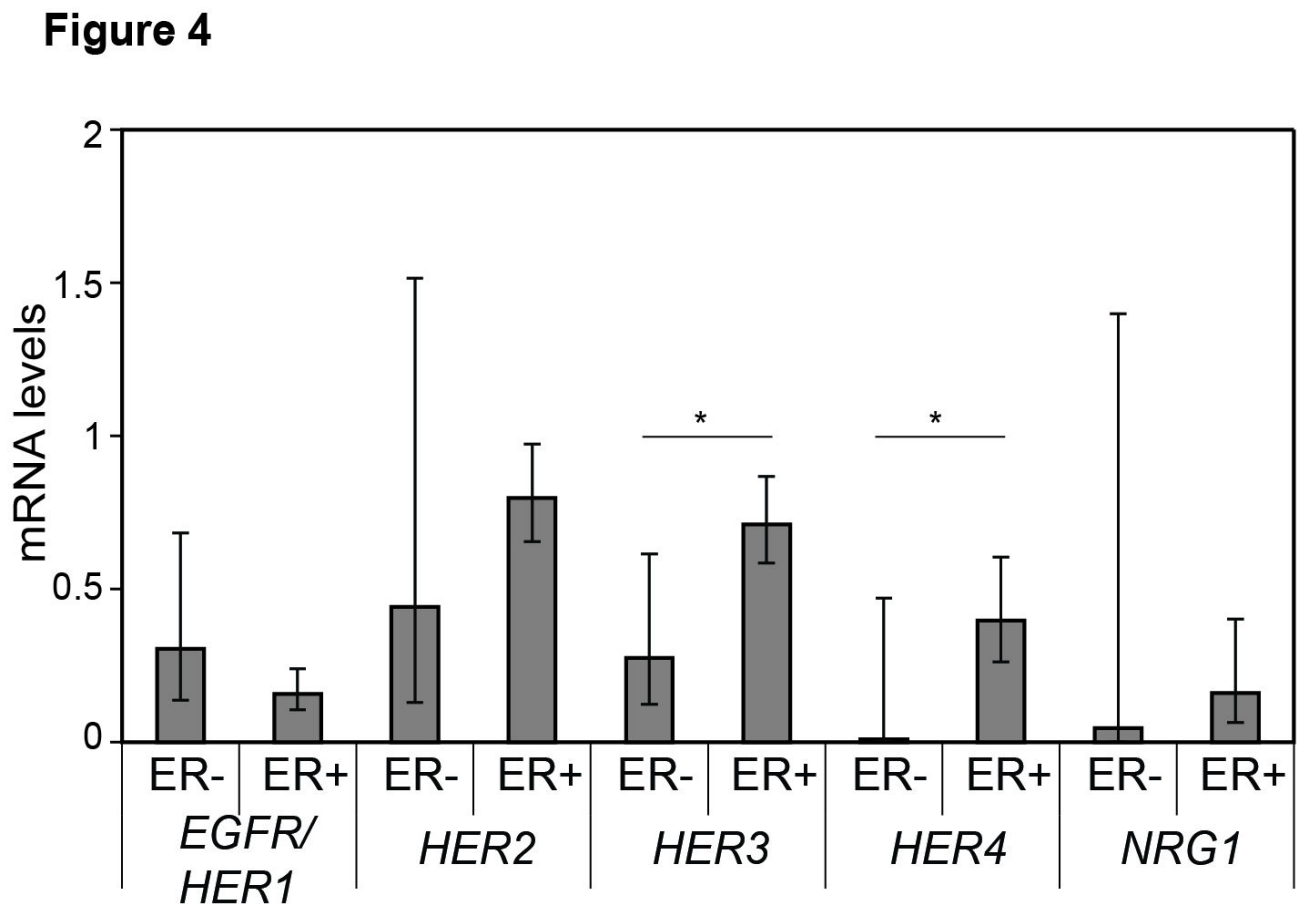
*HER1-4* and *NRG1* intratumoural levels related to estrogen receptor status among postmenopausal women. Geometric mean with 95% confidence intervals of *EGFR/HER1*, *HER2*, *HER3* and *HER4* levels in estrogen receptor positive (ER+) and ER negative (-) HER2 non-amplified tumours from postmenopausal women. Significant differences between ER+ and ER- tumours are presented using the Mann-Whitney U test.

**Figure 5:**
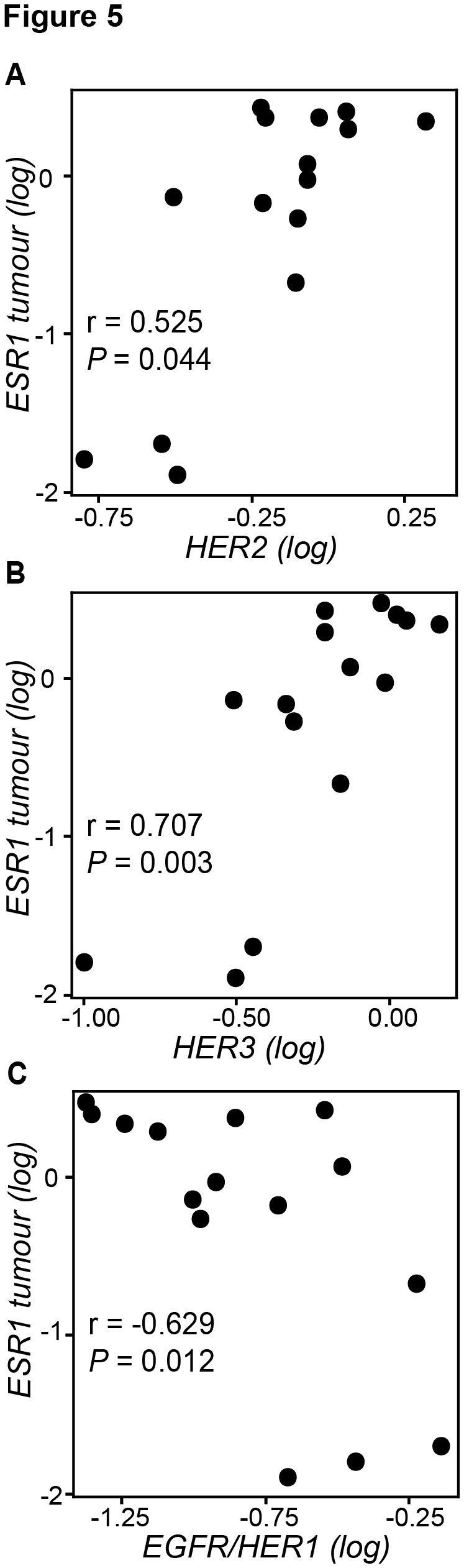
Intratumoural correlations of growth factor receptors with estrogen receptor mRNA levels (*ESR1*). Scatterplots illustrate correlations of *HER2* (A), *HER3* (B) and *EGFR/HER1* (C) with *ESR1* among postmenopausal women. *HER2* amplified tumours are excluded. Significant correlations were evaluated using the Spearman rank (two-tailed) test.

From the subgroup of postmenopausal women, *HER3* (*P*=0.004) and *HER4* (*P*=0.024) were higher among ER+ as compared to ER- tumours (Figure 4). In addition, *HER2* (r=0.525, *P*=0.044, Figure 5A) and *HER3* (r=0.707, *P*=0.003, Figure 5B) correlated positively with *ESR1* expression levels in postmenopausal women, and there was a correlation between intratumoural *HER3* and E2 levels (r=0.544, *P*= 0.007, Table 5). Taken together, these data indicate breast cancer tissue *HER3* and, potentially, *HER2* and *HER4* to be associated with estrogen stimulation.

**Table 5:** Correlations of *HER*-receptors and *NRG1* with estrogen levels in normal tissue, tumour tissue and plasma.

				Tumour				
				*EGFR/HER1*	*HER2*	*HER3*	*HER4*	*NRG1*
Normal tissue	E1		Total^a^	-0.458*	0.084	0.367	0.276	-0.315
			ER+	-0.498*	0.276	0.423	0.370	-0.236
	E2		Total	-0.556**	0.086	0.412	0.403	-0.473*
			ER+	-0.494*	0.187	0.327	0.291	-0.437
	E1S		Total	0.223	0.081	0.114	0.025	0.196
			ER+	0.211	0.049	0.147	0.004	0.158
Tumour	E1		Total	-0.148	0.136	0.200	0.238	-0.106
			ER+	-0.343	0.257	0.354	0.463	-0.214
	E2		Total	-0.633**	0.289	0.544**	0.298	-0.280
			ER+	-0.676**	0.164	0.261	-0.115	-0.542*
	E1S		Total	0.039	0.057	0.133	0.269	0.336
			ER+	-0.029	0.015	0.220	0.394	0.320
Plasma	E1		Total	-0.509*	0.464*	0.336	0.187	-0.331
			ER+	-0.529*	0.245	0.255	0.047	-0.475
	E2		Total	-0.625**	0.285	0.171	0.215	-0.379
			ER+	-0.647**	0.140	0.206	0.194	-0.436
	E1S		Total	-0.417	0.128	0.115	0.059	-0.257
			ER+	-0.321	-0.069	0.034	0.017	-0.228

^a^ The total group of postmenopausal women and the subgroup of ER+ postmenopausal women

Spearman rank correlation (two tailed):

* *P*≤0.05

** *P*≤0.01

In contrast, *EGFR/HER1* expression levels correlated negatively with *ESR1* tumour levels (r=-0.629, *P*=0.012, Figure 5C) in addition to intratumour (r=-0.633, *P*=0.001, Table 5), normal tissue (r=-0.556, *P*=0.005) and plasma (r=-0.625, *P*=0.002) E2 levels. These negative correlations were also significant when restricting the analysis to ER+ tumours only (Table 5). Interestingly, we also observed a trend of negative correlations between tumour *NRG1* levels and estrogens in tissues and plasma where tumour *NRG1* correlated negatively with E2 in normal tissue (r=-0.473, *P*=0.023, Table 5). Moreover, in ER+ tumours we observed significantly negative correlations between intratumour *NRG1* and *ESR1* levels in premenopausal (r=-0.604, *P*=0.017) as well as among postmenopausal women (r=-0.769, *P*=0.003, data not shown). In postmenopausal women harbouring ER+ tumours, we also recorded a negative correlation between *NRG1* and tumour E2 levels (r=-0.542, *P*=0.020, Table 5). These results suggest an association between elevated estradiol and suppression of *EGFR/HER1* and potentially *NRG1* in tumours from postmenopausal women.

## Discussion

In this study we have shown correlations in-between members of the HER-receptor family as well as between members of the HER-receptor family and plasma and tumour tissue estradiol levels in breast cancer patients. The study design provides a unique sample set with matched tumour and normal breast tissue from the same breast together with plasma samples collected synchronously [18]. To our knowledge, this is the first mRNA-expression analysis of all members of the HER-receptor family in addition to the ligand for HER3/4, neuregulin-1 (NRG1) in tumour and normal tissue from breast cancer patients where tissue estrogen levels have been determined in concert.

Experimental studies have shown that resistance to endocrine therapy involve a switch from ER dependent- to growth factor-dependent growth, promoting cross-talks between ER and growth factors, in particular HER2 [21–24]. Notably, among patients with ER positive tumours, overexpression of HER2 has been associated with higher relapse rate during endocrine treatment [25–27]. However, our knowledge about growth factor signalling during development of resistance is limited. EGFR/HER1 and HER2 may play important roles, and these receptors have been found upregulated in response to endocrine treatment in ER positive breast cancer cell lines [28–32]. While clinical evidence is lacking, conflicting evidence have linked HER3 and HER4 status to resistance toward different types of endocrine manipulation *in vitro* [8,33,34]. Moreover, recent findings that combined therapies with trastuzumab and either lapatinib [35] or pertuzumab [36] may improve therapeutic efficacy as compared to trastuzumab monotherapy provides indirect evidence in support of cross-talks between different components of the HER-family.

Clinical studies have reported the benefit of adding HER-targeted drugs to an aromatase inhibitor in ER+ HER2 amplified tumours [3,37], but a potential biological role of the HER-receptor family in tumours not amplified for *HER2* remains poorly understood. In 2009, Johnston et al [2] reported lapatinib to improve therapeutic efficacy of aromatase inhibition in a small subgroup of patients with poor prognosis ER+ tumours harbouring normal *HER2* gene copy numbers. Moreover, in a preclinical study, lapatinib restored endocrine sensitivity in ER+ HER2 non-amplified cells exhibiting endocrine resistance [5]. More recently, in the MAPLE pre-surgical trial, lapatinib was shown to have antiproliferative effects in both HER2 positive and negative breast cancer [38].

Here, we observed increased levels of *HER2*, *HER3* and *HER4* in ER+ HER2 non-amplified tumours compared to normal tissue, implying a role of these receptors in *HER2* non-amplified breast cancer [39]. Conflicting evidence has linked estrogen signalling to HER-receptors transcriptional activity [28,40–45]. Our findings support the existence of a cross-talk between estrogen/ER-signalling and growth factors in ER+ HER2 non-amplified tumour implicating a greater potential of increased growth-factor dependent signalling in tumours compared to normal breast tissue. The strongest difference between normal breast tissue and breast tumours was observed for *HER3*. Together with HER2, HER3 generates the most mitogenic dimer in the HER-family with the capacity to signal both through the mitogen-activated protein kinase (MAPK) pathway for cell proliferation and through the phosphatidylinositol-3´-kinase (PI3K)-Akt pathway for cell survival [46]. HER3 signalling has been shown to play a central role in *HER2* amplified disease [47], however the prognostic value of HER3 in these tumours is unclear [48–50]. Interestingly, HER3 overexpression has been shown to have negative effects on breast cancer survival among patients with *EGFR/HER1* and *HER2* non-amplified tumours [51]. Little is known about the potential direct or indirect effects of estrogens on the transcriptional regulation of HER3, and the mechanisms regulating the activity of HER3 in ER+ HER2 non-amplified tumours should be analysed more in detail. Notably, HER4 has been demonstrated as an estrogen-inducible gene containing estrogen-responsive elements in the promoter, and it also serves as an ER coregulator promoting tumor cell proliferation [45].

Recently, positive correlation between HER2 and ER was observed in HER2 negative tumours [15]. Our results confirm these observations and point out an important difference in biology between *HER2* amplified and non-amplified tumours concerning their relationship to ER. Moreover, our results support the observation that ER- tumours distinguish between HER2 positive- and HER2 negative tumours more clearly than ER+ tumours. All together these findings are important when considering treatment of ER+ breast cancer where HER2 status may not be clearly defined.

NRG1, also known as HRG-beta, is a ligand for HER3 and HER4 and is known to mediate an autocrine signalling loop activating HER3 that stimulates the cell proliferation [52,53]. HER3 is essential for HER2 driven tumourigenesis [47], and patients with NRG1 driven HER2 non-amplified tumours have been suggested to derive clinical benefit from HER2: HER3-directed therapies [53]. On the other hand, *NRG1* has been shown to be silenced by methylation in breast cancers, in which case tumour cells may be deprived of an important growth factor [54]. We did not observe any significant difference in NRG1 mRNA levels between breast cancer and normal tissue; thus, further studies are required to understand NRG1s role or function in endocrine breast cancer and treatment.

We observed lower tumour compared to normal tissue levels of *EGFR/HER1*. Experimental studies have found EGFR/HER1 in general to be low in ER+ breast cancer cell lines probably due to downregulation by estrogens [32,42,55–57]. *EGFR/HER1* is known as an estrogen-responsive gene transcriptionally repressed by estrogens in ER+ breast cancer cells [58,59]. In the clinical setting it has been demonstrated that ER+ tumours have lower levels of EGFR/HER1 protein than ER- tumours [57]. Thus, although our data are based on a relatively small number of patients, they clearly support the *in vitro* findings that *EGFR/HER1* is suppressed by estrogen in tumours, leading to an inverse relationship between ER and *EGFR/HER1*.

The present study has some limitations. Even though robust statistical interpretation has been obtained for several of the correlations analyses, the number of paired normal and tumour samples is limited, especially when we are restricting analyses to postmenopausal women only. We present quantitative data based on mRNA expression levels rather than protein levels. From a clinical point of view protein levels are important since treatment decisions are based on immunohistochemistry data. Correlation between protein and mRNA levels may vary depending on the methods that are used, however *ESR1* mRNA levels are shown to be upregulated in ER+ tumors [17]. Moreover, previous reports that have included analyses of EGFR and HER2 mRNA and protein in the same samples demonstrate high degrees of correlation between the levels of mRNA and protein [48,60,61]. All patients enrolled in this study had tumours distinct palpable in the mastectomy specimen. Since, the tumours may affect the surrounding tissue, it should be noted that the normal tissue was removed from each breast quadrant at significant distance from the primary tumour. While each normal tissue specimen did not undergo histological examination, the breasts had been subject to pre-operative mammography excluding multifocal disease including microcalcifications indicative of cancer *in situ*.

In summary, the present study demonstrates that *EGFR/HER1* is suppressed and negatively associated with estradiol and ER, whereas *HER3*, and potentially *HER2* and *HER4*, are elevated and positively associated with estradiol in *HER2* non-amplified breast tumours from postmenopausal women. Further studies on the effects of endocrine therapy on HER-receptors and ligands should provide more information about the relationship between estrogens and HER-signalling *in vivo.*
